# 

**Published:** 1882-04-20

**Authors:** 


### TABLE OF CONTENTS.

ORIGINAL AND SELECTED ARTICLES.

Some remarks at an Eye Clinic, by A G Hobbs, M D., Professor of Diseases of the Eye, Ear and Throat, in Southern Medical College, Atlanta, Ga., 121. Variola and Vaccinia, by T B Greenley, MD , of Ky., 123. Trade-Marks and Copy-Rights, by R L Hinton, M D,, of Ky., 126 Varicose Veins of the Leg, by B F Duke, M D., of Miss., 127. Information Wanted, by A G Smythe, 128. Case of Abortion with Retained Placenta, 129. Physiological Action of Yerba Santa, 133. Physical and Therapeutical Action of Ergot, 135. Codeia, by J B Garrison, M D., of Ark., 136.

ABSTRACTS AND GLEANINGS.

Sexual Abnormalities, 137. Quinia, 138. Coated Pills, 139. Diagnosis Wanted; Disadvantages of Cod-Liver Oil for Children, 140. Sabal Serrulata; A New Method of Treating Endometritis, 141. Nitrite of Amyl in Convulsions, 142. Chloral and Bromide of Ammonium in Febrile Delirium, 143. Bovine Lymph, 144. Treatment of Ozæna, 145. Danger of Administering Anæsthetics without Witnesses; Case of Poisoning by Atropia, 146. Physicians who do not read, 147. Eczema vs. Vaccination, 148. Real Position of Rotheln, Bubeola or German Measles; The Alum Plug in Uterine Hemorrhage, 149. Antiseptic Inhalation; Explosion of Aqua Ammoniæ; Prophylaxis of Small-Pox, 150.

SCIENTIFIC ITEMS.

Locusts as Food; The Wonders of Paper; Canned Meats and Vegetables, 151. Artificial Leather; Electrical Experiment; Prof. Edward C. [illegible]ickering, 152.

PRACTICAL NOTES AND FORMULÆ.

Rheumatism, Acute and Chronic; Iodoform in Gynecological Practice; Leucorrhœa, 153. Huxley's Theory of Disease; Antiseptic Stimulation in Typhoid; A Dangerous Instrument; Application in Inflamed Conjunctiva, 154. Treatment of Phthisical Cough; Hair Tonic; Borax in Hoarseness; Treatment of Malarial Chill, 155.

EDITORIALS AND MISCELLANEOUS.

Small-Pox in Atlanta; Negro Doctors; Dr. Samuel Gross; Harter's Iron Tonic; Tennessee Medical Society; Johnston's Fluid Beef; New York Code of Ethics; The Pharmaceutical Association of Georgia, 156. Senator Hill; Georgia Medical Association; Crockery and House-Furnishing; Book Notices, 157. Receipts; Special Notices, 160.

